# Use of Colistin and Other Critical Antimicrobials on Pig and Chicken Farms in Southern Vietnam and Its Association with Resistance in Commensal Escherichia coli Bacteria

**DOI:** 10.1128/AEM.00337-16

**Published:** 2016-06-13

**Authors:** Nhung T. Nguyen, Hoa M. Nguyen, Cuong V. Nguyen, Trung V. Nguyen, Men T. Nguyen, Hieu Q. Thai, Mai H. Ho, Guy Thwaites, Hoa T. Ngo, Stephen Baker, Juan Carrique-Mas

**Affiliations:** a Oxford University Clinical Research Unit, Hospital for Tropical Diseases, Ho Chi Minh City, Vietnam; b Department of Medical Microbiology, Academic Medical Center, University of Amsterdam, Amsterdam, The Netherlands; c Sub-Department of Animal Health, My Tho, Tien Giang Province, Vietnam; d Centre for Tropical Medicine, Nuffield Department of Clinical Medicine, Oxford University, Oxford, United Kingdom; FDA Center for Food Safety and Applied Nutrition

## Abstract

Antimicrobial resistance (AMR) is a global health problem, and emerging semi-intensive farming systems in Southeast Asia are major contributors to the AMR burden. We accessed 12 pig and chicken farms at key stages of production in Tien Giang Province, Vietnam, to measure antimicrobial usage and to investigate the prevalence of AMR to five critical antimicrobials (β-lactams, third-generation cephalosporins, quinolones, aminoglycosides, and polymyxins) and their corresponding molecular mechanisms among 180 Escherichia coli isolates. Overall, 94.7 mg (interquartile range [IQR], 65.3 to 151.1) and 563.6 mg (IQR, 398.9 to 943.6) of antimicrobials was used to produce 1 kg (live weight) of chicken and pig, respectively. A median of 3 (out of 8) critical antimicrobials were used on pig farms. E. coli isolates exhibited a high prevalence of resistance to ampicillin (97.8% and 94.4% for chickens and pigs, respectively), ciprofloxacin (73.3% and 21.1%), gentamicin (42.2% and 35.6%), and colistin (22.2% and 24.4%). The prevalence of a recently discovered colistin resistance gene, *mcr-1*, was 19 to 22% and had strong agreement with phenotypic colistin resistance. We conducted plasmid conjugation experiments with 37 *mcr-1* gene-positive E. coli isolates and successfully observed transfer of the gene in 54.0% of isolates through a plasmid of approximately 63 kb, consistent with one recently identified in China. We found no significant correlation between total use of antimicrobials at the farm level and AMR. These data provide additional insight into the role of *mcr-1* in colistin resistance on farms and outline the dynamics of phenotypic and genotypic AMR in semi-intensive farming systems in Vietnam.

**IMPORTANCE** Our study provides accurate baseline information on levels of antimicrobial use, as well as on the dynamics of phenotypic and genotypic resistance for antimicrobials of critical importance among E. coli over the different stages of production in emerging pig and poultry production systems in Vietnam. E. coli isolates showed a high prevalence of resistance (>20%) to critically important antimicrobials, such as colistin, ciprofloxacin, and gentamicin. The underlying genetic mechanisms identified for colistin (the *mcr-1* gene) and quinolone (*gyrA* gene mutations) are likely to play a major role in AMR to those compounds. Conjugation experiments led to the identification of a 63-kb plasmid, similar to one recently identified in China, as the potential carrier of the *mcr-1* gene. These results should encourage greater restrictions of such antimicrobials in Southeast Asian farming systems.

## INTRODUCTION

Antimicrobials are widely used in animal production to prevent and treat disease and to improve growth performance (1). Nearly all antimicrobial classes important for human medicine are also used in animal production (2). The association between antimicrobial use (AMU) and resistance (AMR) in commensal bacteria on poultry and pig farms has been established (3, 4). However, most studies to date have been conducted in the intensive-production systems prevalent in developed countries. In contrast, few data are available from semi-intensive systems prevalent in developing countries, where farmers typically have little access to veterinary support.

Of particular concern is the prevalent use of antimicrobials considered by the World Health Organization (WHO) to be of critical importance for human medicine (5) in Vietnamese pig and poultry farms. These antimicrobials include penicillins, third-generation cephalosporins, quinolones, aminoglycosides, polymyxins, and macrolides (6–8).

Escherichia coli is a commensal organism of the gastrointestinal tract and a widely used marker to monitor AMR in livestock and meat (9, 10). Although a high prevalence of AMR among E. coli strains has been reported on animal farms in Vietnam (7, 11), little is known about the molecular mechanisms of resistance. In E. coli, reduced susceptibility and resistance to quinolones are known to be associated with mutations in the DNA gyrase gene (*gyrA*) (12) and/or the presence of plasmid-mediated quinolone resistance (PMQR) determinants, including the Qnr proteins, the acetylating AAC(6′)-Ib-cr enzyme, and QepA efflux pumps (13). Common aminoglycoside resistance mechanisms among Enterobacteriaceae include expression of adenylyltransferases and phosphoryltransferases encoded by *aadA* and *strA-strB*, respectively, which are located within multidrug resistance (MDR) integrons (14). Very recently, plasmid-mediated colistin resistance, encoded by the *mcr-1* gene, was discovered in E. coli isolates from pigs, chickens, and humans in China. In view of the higher prevalence of positive samples in animal isolates, it is likely that the *mcr-1* resistance mechanism originated in animals and subsequently spread to humans (15).

In this study, we aimed (i) to measure levels of antimicrobial use and AMR and to assess the prevalence of a selection of nine AMR-associated genes encoding resistance to antimicrobials that are commonly used in human health care among commensal E. coli at different stages of production and (ii) to investigate the relationship between phenotypic and genotypic markers of resistance and between AMU and AMR. To address these aims, we conducted a longitudinal study during the production cycle on 12 pig and meat chicken farms typical of emerging semi-intensive systems in the Mekong Delta region of Vietnam.

## MATERIALS AND METHODS

### Selection of study farms.

A total of six chicken (C1 to C6) and six pig (P1 to P6) units representative of emerging semi-industrial production farming systems in the Mekong Delta region of Vietnam were studied. The selected chicken farms met the following criteria: (i) they raised chickens for meat production, (ii) they had between 500 and 10,000 chickens, and (iii) they performed all-in/all-out management (i.e., all chickens were bought and sold at the same time). The selected pig farms met the following criteria: (i) they raised pigs from farrowing to slaughter at any one time on the farm and (ii) they had at least 50 pigs at one any time on the farm.

### Farm sampling.

From each farm, rectal/cloacal swabs were collected from chickens and pigs on three consecutive visits: (i) the day of arrival of chicks from the hatchery (chickens) or 1 to 2 days after farrowing (pigs), (ii) midproduction (chickens, 25 to 30 days old; pigs, 60 to 65 days old); and (iii) immediately before sale (chickens, 45 to 48 days old; pigs, 125 to 130 days old). On the first visit to each farm, 10 randomly selected animals were tagged using leg rings (chickens) or ear notches (pigs). The same animals were sampled on subsequent visits.

### Data collection.

Data on administration of antimicrobials by the farmer and in feed were collected during the farm visits using questionnaires. Data on AMU was defined for two distinct periods: (i) between restocking/birth and the second sampling and (ii) between the second and third samplings. Visits were conducted between November 2013 and June 2014 by trained veterinarians affiliated with the Tien Giang Sub-Department of Animal Health (SDAH).

### Isolation of E. coli.

The rectal/cloacal swabs collected on each visit from the target animals were pooled and tested as one analytical sample. In order to isolate E. coli, samples were streaked directly onto MacConkey agar (Oxoid, United Kingdom) and were subsequently incubated at 37°C overnight. Up to five colonies showing typical E. coli morphology were confirmed using standard biochemical tests (motility, indole, lactose/glucose fermentation, methyl red, citrate, urease, hydrogen sulfide, and gas production).

### Phenotypic testing of AMR.

Colonies confirmed as E. coli were phenotypically tested for their susceptibility to ampicillin (10 μg) and ceftazidime (30 μg) (Oxoid, United Kingdom) by the Kirby-Bauer disk diffusion test. Ciprofloxacin, gentamicin, and colistin resistance was investigated by determining the MIC using Etest (bioMérieux, France). The reference strain E. coli ATCC 25922 was used for quality control purposes. In order to establish the susceptibility status of test strains, the guidelines on breakpoints provided by the Clinical and Laboratory Standards Institute (16) were followed. The strains were considered to have colistin resistance if their MICs were >2 μg/ml, as described by the European Committee on Antimicrobial Susceptibility Testing (17). Colonies that were intermediate in resistance based on the inhibition zone were also regarded as resistant. An MDR organism was defined as a strain that is resistant to at least three different antimicrobial classes.

### PCR amplification of AMR genes and DNA sequencing.

Rapid DNA preparation was performed by a boiling technique that included a heating step (95°C for 15 min) for colonies in a total volume of 200 μl of distilled water, followed by a centrifugation step for the cell suspension. PCR amplification of the quinolone [*qnrA*, *qnrB*, *qnrS*, *qepA*, *aac*(*6′*)-*Ib-cr*, and *gyrA*], aminoglycoside (*aadA* and *strA-strB*), and colistin resistance (*mcr-1*) genes was performed using previously published primer sets (15, 18–20). PCR amplification was performed using a Tprofessional Thermocycler (Biometra, Germany) and BioTaq polymerase (Bioline, United Kingdom). PCR amplicons were examined by electrophoresis and UV visualization on 1.5% agarose gels containing Nancy fluorescent stain (Sigma, Germany). Amplicons produced from all the strains specific for *gyrA* were sequenced with the same primers used for amplification. The forward and reverse strands of all PCR products were sequenced using BigDye terminators on an ABI sequencer (Life Technologies, USA).

### Plasmid conjugation experiment.

The potential transmissibility of the *mcr-1* gene was investigated by performing an *mcr-1*-containing-plasmid conjugation experiment. We independently mated an *mcr-1* plasmid-containing strain (donor) with pansusceptible sodium azide-resistant E. coli J53 (recipient). The bacteria were conjugated for 12 h in Luria-Bertani (LB) broth at 37°C, and transconjugant strains were selected on LB agar plates supplemented with colistin (2 mg/liter) and sodium azide (100 mg/liter). The colonies on each plate were counted, and the number was used to estimate the transfer frequency per donor in successful transfer experiments. Plasmids were extracted from the donors and transconjugants using the method described by Kado and Liu (21). DNA was separated on 0.7% agarose gels and visualized under UV light after staining with ethidium bromide. The approximate size of the plasmid was determined after comparison with E. coli 39R861 containing four plasmids of 147, 63, 36, and 7 kb.

### Data analyses.

The amount of in-feed antimicrobials consumed (in milligrams) to produce 1 kg of live chicken or pig was estimated from the concentration of antimicrobials in each feed product (as indicated on the label) and the feed conversion rate (FCR) (the amount of feed required to produce 1 kg of animal live weight). The FCR values used were 2.85 for chickens and 3.90 for pigs (22, 23). Since some feed products had ambiguous labeling (i.e., the manufacturer indicated that they contained one of two or more different antimicrobials), separate calculations were performed on the assumption that either of the listed antimicrobials was present.

The amount of antimicrobials administered by the farmer (i.e., excluding commercial medicated feeds) to produce 1 kg of live animal was estimated by dividing the total amount of each antimicrobial used (in milligrams) by the estimated body weight (in kilograms) of an animal at the end of its production cycle. Since weight data on chickens and pigs at slaughter were not available, we assumed that the weights of chickens and pigs raised for 2 months and 5 months were 1.5 kg and 92.5 kg, respectively (22, 23).

In order to explore the association between antimicrobial use and AMR, we calculated the Pearson's correlation coefficient between the quantities of antimicrobials used on each farm and the average number of antimicrobials to which E. coli isolates from those farms were resistant for chicken and pig farms separately. The potential association between the use (versus lack of use) of specific antimicrobials during periods 1 and 2 and the observed phenotypic and genotypic AMR (outcome) of isolates recovered on sampling visits 2 and 3 was investigated using univariable risk ratios (RR) for chicken and pig farms. In all cases, the baseline group referred to the prevalence of AMR among isolates from farms that did not use antimicrobials in that period.

The agreement between phenotypic and genotypic resistances for ciprofloxacin, gentamicin, and colistin was determined using the kappa statistic (κ). Statistical analyses were performed using the epicalc and epiR packages in R version 3.0.2 (R Foundation for Statistical Computing).

## RESULTS

### Farms and isolates.

The six chicken farms ranged in size from 500 to 6,000 chickens (mean = 2,572; standard deviation [SD], ±2,318); the six pig farms ranged in size from 73 to 250 pigs (mean = 108; SD, ±61). We isolated a total of 180 E. coli strains (5 isolates/visit) during the routine farm visits.

### Antimicrobials in commercial feed.

All the farms used commercial feed. Totals of 4 and 13 different feed products were found on the chicken and pig farms, respectively. The identity of the feed product (and its antimicrobial composition) could be established for 3 of the chicken and 10 of the pig feed products. A total of two chicken and nine pig products contained at least one antimicrobial (Table 1). However, for 10/11 feed products, the label indicated that the product contained one (only one) of several antimicrobials listed (up to five).

**TABLE 1:** Amounts of antimicrobials present in commercial feeds and estimated consumption of in-feed antimicrobials on each of the 9 farms investigated

Product no.	Period of use^*a*^	Antimicrobial	Concn of antimicrobial in feed (mg/kg live wt)	Estimated consumption of in-feed antimicrobials^*b*^								
				Chicken farms					Pig farms			
				C1	C3	C4	C5	C6	P2	P3	P5	P6
1	1 and 2	Avilamycin OR	10	28.5	28.5	28.5	57.0	28.5				
		Enramycin	10	28.5	28.5	28.5	57.0	28.5				
2	1 and 2	Enramycin	10		28.5	28.5	28.5					
3	1 and 2	Colistin^*c*^ OR	180						702.0			
		Florfenicol OR	30						117.0			
		Kitazamycin OR	110						429.0			
		Tiamulin OR	120						468.0			
		Amoxicillin^*c*^	150						585.0			
4	1 and 2	Avilamycin OR	10						39.0			
		Kitasamycin OR	110						429.0			
		Tiamulin	120						468.0			
5	1 and 2	Tylosin OR	40							156.0		
		Enramycin OR	20							78.0		
		Colistin^*c*^	150							585.0		
6	1 and 2	Chlortetracycline OR	50							195.0		
		Neomycin^*c*^ OR	65							253.5		
		Enramycin OR	20							78.0		
		Virginiamycin	10							39.0		
7	1 and 2	Chlortetracycline OR	50							95.0		
		Virginiamycin OR	10							39.0		
		Lincomycin	20							78.0		
8	1 and 2	Chlortetracycline OR	100								390.0	
		Bacitracin	50								195.0	
9	1	Tylosin OR	40									156.0
		Enramycin OR	20									78.0
		Amoxicillin^*c*^	300									1,170.0
10	1	Tylosin OR	40									156.0
		Enramycin OR	20									78.0
		Colistin^*c*^	150									585.0
11	1 and 2	Chlortetracycline OR	50									195.0
		Lincomycin OR	20									78.0
		Virginiamycin OR	10									39.0
		Enramycin	20									78.0

a Period 1, early phase of production; period 2, late phase of production.

b Details of antimicrobials in feed were not available for farms C2, P1, and P4.

c Antimicrobial considered to be critically important for human medicine according to WHO criteria.

Totals of 2 and 13 different antimicrobials were listed for chicken and pig feed products, respectively. Enramycin and chlortetracycline were the most common antimicrobials present in pig feeds (in five and four products, respectively). Colistin, amoxicillin, and neomycin (all considered to be of critical importance by the WHO) were present in three, two, and one of the pig feed products investigated, respectively. No antimicrobials considered to be of critical importance by the WHO were present in any of the three chicken feeds. Pig feed was supplemented with antimicrobials at significantly higher concentrations (median, 45 mg/kg) than chicken feeds (median, 10 mg/kg) (*P* < 0.001). The median estimated amounts of in-feed antimicrobials used to produce 1 kg (live weight) of chicken and pig were 57.0 (interquartile range [IQR], 28.5 to 57.0) mg and 507.0 (IQR, 312.0 to 877.5) mg, respectively.

### Antimicrobials administered for prophylactic and therapeutic purposes.

In addition to antimicrobials present in feed, all the chickens and pigs were administered antimicrobials by the farmer at least once in their life cycles. Totals of 10 and 15 different antimicrobial products were used on chicken and pig farms, respectively. Fifty percent of the chicken products and 66.7% of the pig products contained two or more antimicrobial compounds among their ingredients. A total of 17 different antimicrobials belonging to eight classes were identified (Table 2). Three chicken products and nine pig products contained antimicrobials considered to be of critical importance for human medicine (neomycin, kanamycin, gentamicin, spiramycin, colistin, and norfloxacin).

**TABLE 2:** Amounts of antimicrobials administered for prophylactic and therapeutic purposes on chicken and pig farms^*a*^

Antimicrobial class	Antimicrobial agent	Amt administered (mg/kg live wt)											
		Chicken farms						Pig farms					
		C1	C2	C3	C4	C5	C6	P1	P2	P3	P4	P5	P6
Aminoglycoside	Neomycin^*b*^							11.7					
	Kanamycin^*b*^									11.1			
	Gentamicin^*b*^					26.7			16.2	6.7		5.9	14.3
β-Lactam	Amoxicillin				40.0	13.3				25.1		22.3	
	Ampicillin									17.8			
Cephalosporin	Cephalexin						5.6						
	Ceftiofur										9.5		
Macrolide	Tylosin	23.0					5.6		12.2				
	Tilmicosin		6.8	10.0									
	Spiramycin^*b*^								12.5				23.8
Phenicol	Chloramphenicol								6.5				
	Florfenicol		11.3	16.7								11.1	
Polypeptide	Colistin^*b*^				5.3	1.8				1.4			3.3
Fluoroquinolone	Enrofloxacin				66.7		11.1			23.3	19.1		40.6
	Norfloxacin^*b*^					53.3					1.8		4.9
Tetracycline	Doxycycline	46.0	5.6	8.3		26.7	5.6					5.5	
	Oxytetracycline							11.7					
Total		69.0	23.7	35.0	112.0	121.8	27.9	23.4	47.4	85.4	30.4	44.8	86.9

a Each line represents one antimicrobial component.

b Antimicrobial considered to be critically important for human medicine according to WHO criteria.

On chicken farms, antimicrobials were administered for prevention of disease in 11 (73.3%) instances and for treatment (of respiratory disease) on four occasions (26.7%). On pig farms, antimicrobials were administered for treatment of respiratory and enteric disease in 18 (66.7%) instances, in 4 (14.8%) instances for disease prevention, and in 5 (18.5%) instances for both purposes. On chicken farms, antimicrobials were administered using water (100% of cases), whereas antimicrobials were administered by injection (93.3% of cases) on pig farms.

The estimated amounts of antimicrobials administered per kilogram of live weight were 52.0 (IQR, 29.7 to 101.2) mg and 46.1 (IQR, 34.0 to 75.9) mg for chickens and pigs, respectively.

### Phenotypic testing for AMR among E. coli isolates.

**FIG 1:**
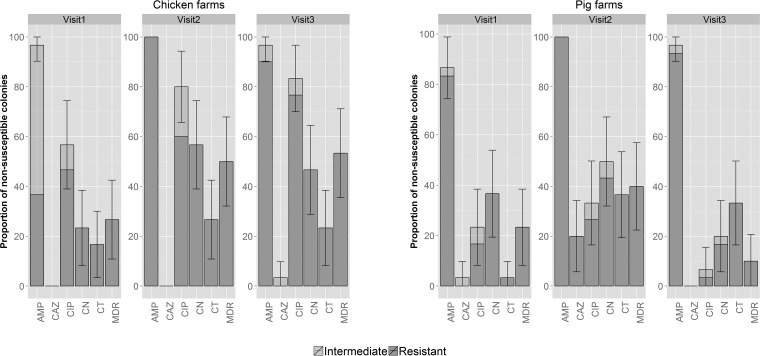
Prevalence of AMR among 180 E. coli isolates (30 isolates/visit/host species) from 12 farms in Tien Giang, Vietnam (2013–2014). The error bars indicate 95% confidence intervals. AMP, ampicillin; CAZ, ceftazidime; CIP, ciprofloxacin; CN, gentamicin; CT, colistin.

The highest prevalence of resistance to specific antimicrobials among E. coli isolates was to ampicillin (97.8% and 94.4% for chickens and pigs, respectively), followed by ciprofloxacin (73.3% and 21.1%), gentamicin (42.2% and 35.6%), colistin (22.2% and 24.4%), and ceftazidime (1.1% and 7.8%) (Fig. 1). The overall prevalence of MDR (defined here as resistance to at least three classes of antimicrobials) among chicken isolates was significantly higher than that among the organisms isolated from pigs (43.3% versus 24.4%, respectively) (*P* = 0.01; Fisher's exact test).

Organisms isolated from chickens exhibited a significantly higher prevalence of resistance to gentamicin at the middle and end of production combined (51.7%) than on the day of arrival (23.3%) (*P* = 0.02; Fisher's exact test). Organisms isolated from pigs exhibited a higher prevalence of colistin resistance at midproduction than at farrowing (36.6% versus 3.3%) (*P* = 0.002; Fisher's exact test). Compared with midproduction, pig isolates from the end of production had a significantly decreased prevalence of resistance to ciprofloxacin (33.3% versus 6.7%) and gentamicin (50.0% versus 20.0%) and a lower rate of MDR isolates (40.0% versus 10.0%) (*P* < 0.03 in all cases; Fisher's exact test).

### PCR screening for AMR-associated genes.

**FIG 2:**
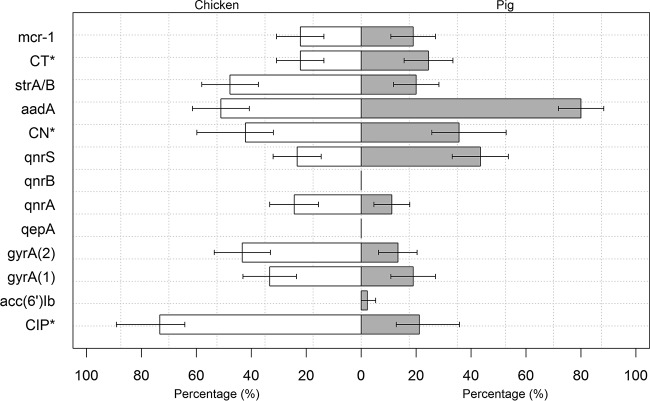
Prevalence of antimicrobial resistance genes among 180 E. coli isolates recovered from 12 farms in Tien Giang, Vietnam (2013–2014). *, phenotypic prevalence of antimicrobial resistance. (1), single mutation; (2), double mutation. The error bars indicate the 95% CI.

Quinolone resistance in E. coli is determined predominantly by mutations at codons 83 and 87 in the *gyrA* gene. A total of 33.3% of chicken isolates and 18.3% of pig isolates had a single mutation in the *gyrA* gene. Double mutations were found in 43.3% of chicken isolates and 13.3% of pig isolates. A total of 24.4% of chicken and 11.1% of pig E. coli isolates produced amplicons for the PMQR gene *qnrA*; 23.3% of chicken isolates and 43.3% of pig isolates were positive for *qnrS*. We detected the *aac*(*6′*)-*Ib-cr* gene in two pig isolates. None of the isolates from chicken or pig harbored either the *qnrB* or the *qepA* gene (Fig. 2). Isolates from chickens had a higher prevalence of *gyrA* double mutations (*P* < 0.001; Fisher's exact test) and *qnrA* (*P* = 0.03; Fisher's exact test) than isolates from pigs. In contrast, isolates from pigs had a higher prevalence of the *qnrS* gene than isolates from chickens (*P* = 0.007; Fisher's exact test). Among the organisms isolated from pigs, the prevalence of double mutations in the *gyrA* gene was higher among isolates from midproduction (26.6%) than among the isolates from the end of production (3.3%) (*P* = 0.03; Fisher's exact test). There was no difference in the prevalence of PMQR determinants between different stages of production.

The prevalence of the *aadA* gene (encoding aminoglycoside resistance) was higher among pig isolates (80.0%) than among chicken isolates (51.1%) (*P* < 0.001; Fisher's exact test). In contrast, the prevalence of the *strA-strB* genes was lower in pig isolates (20.0%) than in chicken isolates (47.8%) (*P* = 0.001; Fisher's exact test). The prevalence of *strA-strB* was higher among chicken isolates from midproduction (60.0%) than among isolates from 1-day-old chicks (26.7%) (*P* = 0.02; Fisher's exact test). In contrast, no changes in the prevalence of *strA-strB* was observed between the stages of pig production.

The prevalences of the recently discovered plasmid-mediated colistin resistance gene *mcr-1* were 18.9% and 22.2% in pig and chicken E. coli isolates, respectively (Fig. 2). The prevalence of *mcr-1* did not change significantly during the production cycle. Among 20 and 17 *mcr-1*-positive E. coli isolates from chickens and pigs, respectively, colistin resistance could be successfully transferred by conjugation in 70.0% and 35.3% of the isolates. The estimated transfer frequency in successful experiments was 10^−1^ to 10^−3^ cells per donor cell. All transconjugants acquired one plasmid approximately 63 kb in size. Furthermore, 7 of the 20 transconjugants contained additional plasmids that were <63 kb or >100 kb in size.

### Relationship between phenotypic and genotypic resistance.

Double mutations in the *gyrA* gene exhibited the highest agreement with ciprofloxacin resistance for both chicken and pig isolates (κ = 0.43 and 0.73, respectively) (Table 3). A single *gyrA* mutation was associated with low levels of resistance to ciprofloxacin (median MIC, 3 [IQR, 1.0 to 32] μg/ml in chicken and 0.75 [IQR, 0.19 to 3] μg/ml in pig isolates), and double mutations were associated with high levels of resistance (median MIC, 32 μg/ml [IQR, 24 to 32 μg/ml and 4 to 16 μg/ml for chicken and pig isolates, respectively]). Amplification of PMQR had a lower agreement with phenotypic ciprofloxacin resistance (κ < 0.2) in all isolates from chickens and pigs.

**TABLE 3:** Estimation of the level of agreement between resistance phenotypes and genotypes in E. coli isolates

Antimicrobial	Gene(s) carried	Chicken^*a*^						Pig^*a*^					
		κ	a	b	c	d	*P* value	κ	a	b	c	d	*P* value
Ciprofloxacin	*qnrA*	0.07	20	48	4	18	0.150	−0.07	2	17	8	63	0.835
	*qnrB*	NC	0	66	0	24	NC	NC	0	19	0	71	NC
	*qnrS*	0.02	19	50	5	16	0.368	0.12	6	13	33	38	0.127
	*aac*(*6′*)-*Ib-cr*	NC	0	66	0	24	NC	0.15	2	17	0	71	0.055
	*qepA*	NC	0	66	0	24	NC	NC	0	19	0	71	NC
	*gyrA*_single_	0.01	22	44	8	16	0.50	0.24	7	12	10	61	0.012
	*gyrA*_double_	0.43	39	27	0	24	<0.001	0.73	12	7	0	71	<0.001
Gentamicin	*aadA*	0.33	28	11	18	33	<0.001	0.13	29	3	43	15	0.030
	*strA-strB*	0.26	26	13	17	34	<0.001	0.19	10	22	8	50	0.011
Colistin	*mcr-1*	1.0	20	0	0	70	<0.001	0.84	17	5	0	68	<0.001

a a, positive genotype, positive phenotype; b, negative genotype, positive phenotype; c, positive genotype, negative phenotype; d, negative genotype, negative phenotype; NC, not calculated.

Among the E. coli organisms isolated from chickens, the presence of *aadA* and *strA-strB* exhibited a reasonable agreement with gentamicin resistance (κ *=* 0.33 and 0.26, respectively) (Table 3). Among pig isolates, the presence of *strA-strB* had the strongest agreement with gentamicin resistance (κ *=* 0.19).

The presence of *mcr-1* had very strong agreement with colistin resistance in both chicken (κ = 1.0) and pig (κ = 0.84) isolates. Among the *mcr-1*-positive strains, the median MIC of colistin was 4 μg/ml (IQR, 3 to 4 μg/ml and 4 to 6 μg/ml for chicken and pig isolates, respectively).

### Association between antimicrobial use and antimicrobial resistance.

We found no correlation between total use of antimicrobials at the farm level and AMR (calculated as the average number of antimicrobials against which E. coli isolates were resistant) (*P* > 0.83). However, the use of quinolones and cephalosporins in the period between birth and midproduction was statistically associated with ciprofloxacin resistance (RR = 9.0 and 5.0, respectively) and colistin resistance (RR = 11.0 and 4.17, respectively) in midproduction on pig farms (Table 4). Cephalosporin use in the first period of production was strongly associated with detection of the *strA-strB* genes in isolates from the second sampling (RR = 5.0). Mutations in *gyrA* among E. coli isolates were strongly associated with the use of phenicols (RR = 20.0), tetracyclines (RR = 10.0), and β-lactams (RR = 5.0) throughout the whole production cycle and with the use of quinolones (RR = 4.5) and cephalosporins (RR = 4.17) in the first period. No association between antimicrobial use and resistance was observed on chicken farms.

**TABLE 4:** Associations between antimicrobial use and phenotypic and genotypic resistance among E. coli isolates from pigs

Exposure	No. of farms using antimicrobial	Outcome^*a*^	RR^*b*^	95% CI^*c*^	*P* value
Exposure, antimicrobial use in period 1; outcome, resistance in second sampling					
Quinolones	3	CT	11.0	2.71–44.66	<0.001
Cephalosporins	1	CT	4.17	1.49–11.71	0.007
Quinolones	3	CIP	9.0	1.84–44.07	0.007
Cephalosporins	1	CIP	5.0	1.71–14.6	0.003
Cephalosporins	1	*strA-strB*	5.0	1.34–18.61	0.016
Quinolones	3	*gyrA*^*d*^	4.50	1.23–16.46	0.023
Cephalosporins	1	*gyrA*^*d*^	4.17	1.49–11.71	0.007
Exposure, antimicrobial use over whole production period; outcome, resistance in third sampling					
Tetracyclines	1	*gyrA*^*d*^	10.0	2.71–36.96	<0.001
β-Lactams	2	*gyrA*^*d*^	5.0	1.02–24.52	0.047
Phenicols	1	*gyrA*^*d*^	20.0	4.38–91.42	<0.001

a CIP, ciprofloxacin; CT, colistin.

b Only significant (*P* < 0.05) risk ratios are presented.

c CI, confidence interval.

d Single or double mutation.

## DISCUSSION

There are very few published longitudinal studies quantifying AMU and describing AMR in poultry and pigs in developing countries (3, 4). Our work represents an important contribution toward understanding the interplay between AMU and AMR on farms in Southeast Asia.

Our results indicate that comparable quantities of antimicrobials were administered by farmers to produce chicken meat and pig meat (52.0 mg per kg live weight versus 46.1 mg, respectively). However, consumption of antimicrobials in feed was considerably higher in pigs than in chickens, resulting in overall greater amounts of antimicrobials consumed by pigs (563.6 mg per kg live weight) than chickens (94.7 mg per kg live weight). Critically important medicines for humans, such as colistin, neomycin, gentamicin, kanamycin, and norfloxacin, were also used on the studied farms. On pig farms, a median of 3 (out of 8) critical antimicrobials were used to raise the animals. A previous study in Vietnam showed much larger amounts of antimicrobials were administered by farmers on meat chicken farms (470.4 mg per chicken produced, equivalent to 276.7 mg per kg, assuming an average weight of 1.7 kg) (6). These differences may reflect management practices related to intensification of farming systems. In this study, farms were much larger (mean, 2,572 chickens per farm), whereas in our previous study, all the farms had <2,000 chickens. The same study showed that larger production units used up to five times less antimicrobials per time unit than smaller units (6). Antimicrobials administered to chickens were mostly used to prevent disease (73.3%); in contrast, most antimicrobials administered to pigs were used for disease treatment (66.7%). Chickens were often administered antimicrobials through water or feed, whereas pigs were administered antimicrobials mostly through injection. This may have resulted in higher levels of AMR in the gastrointestinal microbiota in the chicken species.

We found higher prevalences of resistance to ciprofloxacin and gentamicin among E. coli isolates than those in previous studies in the region (73.3% and 42.2% compared to 21.0 to 24.2% and 10.8 to 15.0%, respectively) (7, 11). Our results show that mutations in the *gyrA* sequence play a key role in the development of resistance to quinolone antimicrobials, whereas the PMQR-associated mechanisms investigated appeared to be less common (12, 24). The presence of both *aadA* and *strA-strB* was most commonly associated with gentamicin resistance in chickens and pigs, respectively. Published studies have shown an association between these genes and resistance to streptomycin and other aminoglycosides among E. coli isolates from poultry and pigs (25, 26).

A particular concern is the high prevalence (22% to 25%) of colistin (a polymyxin antimicrobial) resistance among pig and chicken E. coli isolates. Colistin is regarded as a last-line antimicrobial for the treatment of severe human infection cause by MDR Gram-negative bacteria. Historically most colistin resistance mechanisms have involved chromosomal mutations (27). More recently, studies from Europe and Asia have reported the emergence of the *mcr-1* gene in Enterobacteriaceae of chicken and pig origins (15, 28, 29). Our results indicated a prevalence of the plasmid-mediated *mcr-1* gene in E. coli isolates similar to those reported in the Chinese study. We also observed very strong agreement between colistin phenotypic resistance and the presence of the *mcr-1* gene in both chicken and pig isolates. The plasmids containing the *mcr-1* gene were of similar size (∼63 kb) to those reported in China (15) and showed a very high *in vitro* transfer rate between E. coli strains. These results suggest that this *mcr-1*-containing plasmid is probably widespread globally by now. Our findings indicated that colistin is commonly used on chicken and pig farms (on 4/12 farms as a prophylactic/therapeutic drug), as well as potentially in feed on 3 pig farms, as has been shown in previous studies in Vietnam (6, 8). We did not, however, observe an increased prevalence of colistin resistance among the four farms where the farmers had administered colistin. In addition, ∼20% of the isolates from 1-day-old chicks tested positive, suggesting vertical transmission from breeder flocks and the capacity of colistin-resistant strains to survive the hatchery process. Given the ambiguous labeling of the feed products with regard to their antimicrobial contents, it is not possible to determine whether colistin was also used in the feed on three other farms. Our findings suggest that the use of quinolones and cephalosporins may select for colistin resistance in pigs, although the reasons for this are currently unknown.

Our results reflect the complexity of AMR and the difficulties in accurately establishing the specific genetic mechanisms underlying specific types of AMR due to the multitude of potential genetic mechanisms. Especially in the cases of colistin (the *mcr-1* gene) and quinolones (*gyrA* gene mutations), we believe that the genetic mechanisms identified are likely to make major contributions to AMR to those compounds. In contrast, the genes assayed (*aadA* and *strA-strB* genes) seemed to have made a smaller contribution to gentamicin resistance.

We did not find an overall relationship between total antimicrobial use and AMR on farms. In contrast, we found some unexplained univariable relationships between the use of certain antimicrobials and certain phenotypic and genotypic AMR patterns on pig farms. This has to be interpreted with caution, especially given the relatively small sample size of our study (6 chicken and 6 pig farms), all subjected to considerable AMR selection pressure, and the potential existence of confounding factors that were not included in the analyses. Unfortunately, the fact that on all the farms antimicrobials were used intensively (i.e., no negative controls were available) makes it difficult to elucidate this relationship. Also, on all the farms, antimicrobials were present in commercial feeds, but it was not possible to determine exactly which antimicrobials are likely to confound this association. In addition, AMR bacteria may also be acquired from external sources and potentially transfer to current animals from animals kept in the building during the previous cycle, (carryover). Because of the impossibility of anticipating the behavior of farmers, we believe that such studies should preferably be performed in experimental-farm settings, where antimicrobial exposure (i.e., quinolones and macrolides) is well controlled.

Among E. coli isolates from pigs, lower prevalences of AMR (for ciprofloxacin and gentamicin) and MDR were found in finishers than in younger pigs. It has been suggested that this may be a reflection of the fitness cost of resistant organisms in the intestinal tract (30). For chickens, an overall lower prevalence of AMR and MDR was found among chicks sampled on arrival at the farm, and an increase was detected for all antimicrobials during production. However, levels of full resistance to ciprofloxacin and ampicillin in 1-day-old chicks were particularly high (46.7% and 36.7%, respectively). Unfortunately, information about potential antimicrobial use in hatcheries was not gathered. On poultry farms, resistant bacteria may be introduced through vertical transmission from parental flocks or contamination in the hatchery environment (31). The presence of resistance in newborn piglets is likely to reflect lateral transmission from the sows (32). In addition to AMU, husbandry practices, inadequate cleaning and disinfection, and the types of feed used may all contribute to AMR (7, 32).

In spite of the small sample size (12 farms), our study provides accurate baseline information on AMU, as well as on the dynamics of phenotypic and genotypic resistance among E. coli isolates over the different stages of production in emerging pig and poultry production systems in southern Vietnam. We recommend that future research focus on the simultaneous detection of large numbers of AMR genes of greater concern, especially those coding for resistance to critically important antimicrobials that are carried on mobile genetic elements. We also recommend that longitudinal studies capture quantitative changes in the resistome in the sample matrix (as opposed to individual colonies). Although such technologies are currently very costly, they will probably become affordable in the near future. E. coli showed a high prevalence of resistance to a last-line antimicrobial (colistin), as well as antimicrobials (ciprofloxacin and gentamicin) critically important for human medicine. We strongly recommend that these important antimicrobials be restricted to treatment of clinical disease and not be used for prophylaxis.
